# Potential amelioration of liver function by low-dose tolvaptan in heart failure patients

**DOI:** 10.1016/j.toxrep.2025.102009

**Published:** 2025-03-24

**Authors:** Yasuaki Mino, Kohei Hoshikawa, Takafumi Naito, Shunta Akutsu, Yumi Imoto, Emi Nakatsugawa, Masao Saotome, Yuichiro Maekawa, Junichi Kawakami

**Affiliations:** a Department of Hospital Pharmacy and Hamamatsu University School of Medicine, 1-20-1 Handayama, Chuo-ku, Hamamatsu 431-3192, Japan; b Third Department of Internal Medicine, Hamamatsu University School of Medicine, 1-20-1 Handayama, Chuo-ku, Hamamatsu 431-3192, Japan; c Department of Pharmacy, Shinshu University Hospital, 3-1-1 Asahi, Matsumoto, Nagano 390-8621, Japan

**Keywords:** Tolvaptan, Liver injury, Pharmacokinetics, DM-4103, DM-4107

## Abstract

**Aim:**

This study aimed to evaluate the relationships between the pharmacokinetics of tolvaptan and its metabolites (DM-4103 and DM-4107) and liver injury in heart failure patients, using relevant laboratory test values and markers of hepatocyte injury and biliary cholestasis.

**Method:**

The plasma concentrations of tolvaptan, DM-4103, and DM-4107 were determined using LC-MS/MS in 51 Japanese heart failure patients. The relationships between the concentrations and the N-terminal fragment of pro-B-type natriuretic peptide (NT-proBNP), AST, and ALT were assessed. K18 and glutamate dehydrogenase as a marker of liver injury and CP-I and CP-III as indicators of OATP activity were also determined.

**Results:**

The median concentrations of tolvaptan, DM-4103, and DM-4107 were 16.2, 287, and 38.0 ng/mL, respectively. AST, ALT, and T-Bil were significantly decreased after tolvaptan administration. They were negatively correlated with tolvaptan concentration. AST was also negatively correlated with DM-4107 concentration. CP-III was positively correlated with DM-4103 concentration; however, CP-I was negatively correlated with DM-4103 concentration. K18 and glutamate dehydrogenase were not correlated with tolvaptan concentration.

**Conclusion:**

Low-dose tolvaptan did not cause liver injury. Pharmacokinetics of tolvaptan may be associated with potential amelioration of liver function in heart failure patients.

## Highlights

• Tolvaptan concentration was inversely correlated with liver function markers.
• Bilirubin was lower in patients with OATP1B3c.1683–5676 A>G.
• Coproporphyrin was not correlated with tolvaptan pharmacokinetics.
• Low-dose tolvaptan improved liver function, contrary to previous reports.
• Specific mechanism underlying the improvement was not determined.

## Graphical Abstract

**Figure:**
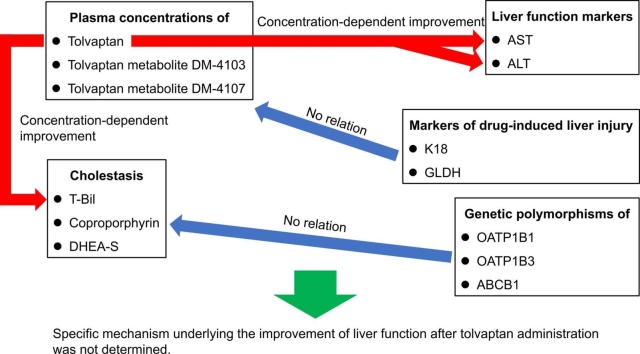

## 1 Introduction

Tolvaptan is an oral selective vasopressin V_2_ receptor antagonist that increases water excretion without loss of electrolytes by suppressing the expression of aquaporin-2 water channels along the renal collecting ducts [1]. The US Food and Drug Administration issued an alert on the potentially fatal liver injury attributed to the high-dose tolvaptan regimen for autosomal dominant polycystic kidney disease [2]. Tolvaptan has a warning of hepatotoxicity even for the low-dose treatment for volume overload related to heart failure in the package insert [3], [4]. Tolvaptan-induced hepatic injury, the factors of which have not yet been fully identified, has been associated with idiosyncratic reaction [5].

The tolvaptan metabolites DM-4103 and DM-4107 have been recently reported to inhibit human hepatic bile acid transporters [6]. Severe hepatic disorders caused by tolvaptan often involve an elevation of serum total bilirubin [3]. The main metabolite, DM-4103, is an inhibitor of organic anion transporting polypeptide (OATP) 1B1 and organic anion transporter 3 *in vitro*. However, with a long elimination half-life of approximately 180 hours, it did not inhibit OATP1B1 in clinical settings [7]. Bilirubin, which is recognized by ATP-binding cassette subfamily B member 1 (ABCB1) and taken up into hepatocytes by OATP1B1 and 1B3, is a known antioxidant cytoprotectant [2]. Although the tolvaptan metabolites have the potential to cause liver injury through transporter inhibition, their effects on liver and biliary functions have not been elucidated in heart failure patients. No information is available concerning the relationship between plasma tolvaptan pharmacokinetics and hepatic disorders in heart failure patients.

Coproporphyrin (CP)-I and III, which are well-known substrates of OATP1B1 and 1B3, are endogenously generated from heme biosynthesis [8]. Plasma CP-I and III are not elevated by hepatocellular disorders [9], unlike serum total bilirubin. The coproporphyrins have more specificity towards OATP activity than total bilirubin [10], [11]. Plasma dehydroepiandrosterone sulfate (DHEA-S), which is a circulating steroid hormone, is also a useful endogenous probe for evaluating the influx activity of OATPs [12]. There are some potentially functional genetic variants of OATP1B1 and 1B3 [13], [14], however, their influence on the plasma levels of the OATP substrates remains to be clarified.

Plasma markers of drug-induced liver injury have been used for diagnostic and predictive purposes [15]. Plasma cytokeratin 18 (K18), which is a cytoskeleton protein released by necrosis and apoptosis of hepatocytes, is a prominent marker for the early detection of drug-induced liver injury with low interindividual variability [16]. Plasma glutamate dehydrogenase (GLDH) is another mechanistic marker of liver injury that exists in mitochondria and reflects only the necrosis of hepatocytes [17]. These markers have an advantage over serum aspartate aminotransferase (AST) and alanine aminotransferase (ALT) for predicting the risk of liver dysfunction at an early stage.

This study aimed to assess plasma exposures of tolvaptan and its metabolites, and their impact on liver and biliary functions in heart failure patients, using relevant laboratory test values and markers of hepatocyte injury and biliary cholestasis.

## 2 Materials and methods

### 2.1 Ethics

This study was conducted according to the Helsinki Declaration of 1964 and its later amendments. The protocol was approved by the Ethics Committee of Hamamatsu University School of Medicine (20−105). The present study was designed as a single-institutional observation study at Hamamatsu University Hospital (Hamamatsu, Japan) and is registered in the University Hospital Medical Information Network-Clinical Trial Registry (UMIN000041334). All of the recruited patients were fully briefed about the research details and provided written informed consent for participation in the study.

### 2.2 Patients and blood sampling

Japanese inpatients (*n* = 95) administered a tolvaptan tablet (Samsca, Otsuka Pharmaceutical Co., Ltd., Tokyo, Japan) for heart failure accompanied by fluid retention once daily after breakfast were recruited. We excluded 13 patients due to high serum total bilirubin (> 2.0 mg/dL) before starting tolvaptan treatment. Other exclusion criteria were the following: patients who were suspected of being low adherers to tolvaptan according to pharmacist monitoring and treatment records (*n* = 2); were undergoing dialysis (*n* = 4); had liver and bile duct cancer or severe hepatic disease (*n* = 5); had active infectious disease (*n* = 6); had a change of prescription for amiodarone or statins (*n* = 7); or were being treated together with potent inducers and inhibitors of CYP3A, ABCB1, OATP1B1, and OATP1B3 such as carbamazepine, cyclosporine, macrolide antibiotics, rifampicin, tacrolimus, azole antifungal agents, and verapamil (*n* = 7) [19]. A total of 51 patients were therefore enrolled. Their blood samples were collected immediately prior to breakfast on the day of the 7th and subsequent doses of tolvaptan.

### 2.3 Quantification of tolvaptan and its metabolites in plasma

Tolvaptan, DM-4103, and DM-4107 (Otsuka Pharmaceutical Co., Ltd., Tokyo), and tolvaptan-*d7* (Medical Isotopes, Inc., Pelham, NH, USA) as an internal standard (IS) in plasma were deproteinized with only acetonitrile. The analytes were quantified by a liquid chromatography system (Nexera X2, Shimadzu Corporation, Kyoto, Japan) coupled to a triple quadrupole tandem mass spectrometer (LCMS-8050, Shimadzu Corporation) equipped with an electrospray probe as previously described [18]. The mixture of acetonitrile and 5 mmol/L ammonium acetate at pH 3.5 was passed through a TSKgel ODS-100V (3 µm, 75 mm length × 2.0 mm I.D., Tosoh, Tokyo) column set at 40°C with a 6-minute linear gradient from 40 % to 70 % acetonitrile at a flow rate of 0.2 mL/min. Tolvaptan, DM-4103, DM-4107, and IS were measured at the respective transitions of *m/z* 448.90–252.15, 477.05–433.05, 479.05–224.05, and 455.90–259.25. The lower limits of quantification (LLOQs) and calibration ranges of tolvaptan, DM-4103, and DM-4107 were 2, 20, and 2 ng/mL, and 2–500, 20–5000, and 2–500 ng/mL, respectively. The intra- and inter-assay accuracies of the analytes were 101.1–106.0 % and 99.0–108.8 %, while their imprecisions were 1.9–3.1 % and 2.0–4.0 %, respectively.

### 2.4 Serum liver and biliary function test results

Serum laboratory test values were extracted from medical records. The post-treatment values, which were defined as the median values over the span of 14 days after the blood sampling, were employed for analysis unless otherwise stated. The baseline values were obtained at a point just before initiation of tolvaptan treatment. Changes in serum liver and biliary function test results following tolvaptan treatment were analyzed.

### 2.5 Quantification of CP-I and III and DHEA-S in plasma

CP-I and III (Frontier Scientific, Inc., Logan, UT, USA), and CP-I-^15^N_4_ (Toronto Research Chemicals Inc., Ontario, Canada) as an IS in plasma were deproteinized with acetonitrile and extracted with dichloromethane. The analytes were quantified by a liquid chromatograph coupled to a tandem mass spectrometer using a TSKgel ODS-100V (3 µm, 150 mm length × 2.0 mm I.D., Tosoh) column set at 40°C. They were eluted with acetonitrile-water (45:55, v/v) containing 0.05 % formic acid at a flow rate of 0.2 mL/min with a total run time of 10 minutes. CP-I, III, and IS were determined at their respective transitions of *m/z* 655.30–596.35, 655.00–596.35, and 659.00–600.35. The LLOQs and calibration ranges of CP-I and III were both 0.1 ng/mL and 0.1–12 ng/mL, respectively. The intra- and inter-assay accuracies of CP-I and III were 99.2–100.9 % and 98.9–102.6 %, and 96.7–100.0 % and 95.1–100.4 %, while their imprecisions were 1.7–12.5 % and 1.4–6.8 %, and 1.9–6.3 % and 2.0–6.6 %, respectively. Plasma DHEA-S was measured using a DHEA-S ELISA Kit (#ab108669, Abcam, Cambridge, UK). The LLOQ of plasma DHEA-S was 0.04 μg/mL.

### 2.6 OATP1B1 and 1B3 genotyping

Genomic DNA samples extracted from peripheral blood using a DNA Extractor WB Kit (FUJIFILM Wako Pure Chemical Corporation, Osaka, Japan) were stored at −20°C until analysis. Potentially functional single nucleotide polymorphisms (SNPs) including rs2306283 (*OATP1B1c.388 A>G*), rs4149056 (*OATP1B1c.521T > C*), rs4149015 (*OATP1B1 g.–11187G>A*), rs4149117 (*OATP1B3c.334T > G*), rs7311358 (*OATP1B3c.699 G>A*), rs11045585 (*OATP1B3c.1683–5676 A>G*), and rs1045642 (*ABCB1 c.3435 C>T*) were evaluated using a TaqMan real-time polymerase chain reaction method (Thermo Fisher Scientific, Waltham, MA, USA).

### 2.7 Plasma markers for the early detection of drug-induced liver injury

Plasma K18 was measured by enzyme-linked immunosorbent assay (ELISA) using a Human Cytokeratin 18 ELISA Kit (#ab227896, Abcam, Cambridge, UK). Plasma GLDH was quantified colorimetrically by an enzymatic method using a Glutamate Dehydrogenase Activity Assay Kit (#ab102527, Abcam, Cambridge, UK). The LLOQs of plasma K18 and GLDH were 3.4 pg/mL and 1.0 U/L, respectively.

### 2.8 Statistical analysis

All data were analyzed using SPSS (version 29.0.20.0, IBM SPSS Statistics, Chicago, IL, USA). Values below the LLOQ were substituted by the LLOQ before subsequent statistical analyses. Difference in continuous variables among three groups were assessed by the Kruskal-Wallis test. Continuous values between unpaired and paired groups were analyzed by the Mann-Whitney *U* test and the Wilcoxon signed rank test, respectively. The Spearman rank correlation coefficient, *r*_*s*_, was employed to test the null hypothesis of no association between continuous values. Descriptive statistics are expressed as the median and interquartile range (IQR). Statistical significance was defined as a two-sided *P*-value < 0.05.

## 3 Results

### 3.1 Descriptive statistics of patients

The clinical characteristics of the 51 Japanese patients are summarized in Table 1. The patients with mild to severe heart failure based on the New York Heart Association (NYHA) classification were treated with tolvaptan 3.75–15 mg daily. Their plasma N-terminal fragment of pro-B-type natriuretic peptide (NT-proBNP) concentrations were 13.3 ng/mL as the median. Twenty-two (43.1 %) and 7 (13.7 %) patients were cotreated with statins and amiodarone, respectively.

**Table 1:** Clinical characteristics of the enrolled patient.

Patient characteristics and baseline serum laboratory test values	
Gender, male/female	27/24
Age, years	77 (71–83)
Body weight, kg	53.5 (47.6–63.5)
Acute/chronic heart failure	21/30
NYHA classification, Ⅱ/Ⅲ/Ⅳ	17/23/11
Plasma NT-proBNP, ng/mL	13.3 (7.2–21.7)
Underlying heart disease	
Ischemic heart disease	21
Valvular heart disease	26
Disease complication	
Atrial fibrillation	21
Diabetes mellitus	24
Hypertension	27
Concomitant drugs	
Loop diuretic	47
Statins	22
Amiodarone	7
Sodium, mEq/L	141 (139–143)
Potassium, mEq/L	4.2 (4.0–4.6)
Chloride, mEq/L	104 (102–107)
Blood urea nitrogen, mg/dL	25 (19–31)
Creatinine, mg/dL	1.4 (0.9–1.6)
C-reactive protein, mg/dL	0.50 (0.20–1.60)

Data are expressed as the number of patients or the median with interquartile range in parentheses.

NT-proBNP, N-terminal fragment of pro-B-type natriuretic peptide; NYHA, New York Heart Association.

### 3.2 Pharmacokinetics of tolvaptan and its metabolites

The median and IQR for plasma concentrations of tolvaptan, DM-4103, and DM-4107 were 16.2 and 7.7–26.8, 287 and 157–485, and 38.0 and 27.9–62.7 ng/mL, respectively.

### 3.3 Influence of tolvaptan treatment on serum liver and biliary function test results

**Fig. 1:**
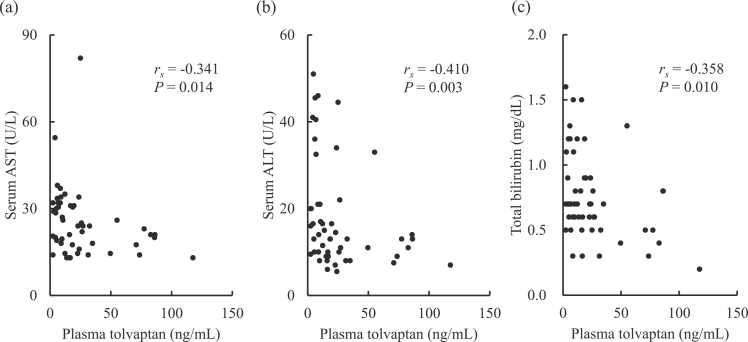
Correlations between serum liver and biliary function test results and plasma concentration of tolvaptan in heart failure patients. Plasma tolvaptan versus serum (a) AST, (b) ALT, and (c) total bilirubin. The correlations were evaluated using the Spearman test. ALT, alanine aminotransferase; AST, aspartate aminotransferase.

**Fig. 2:**
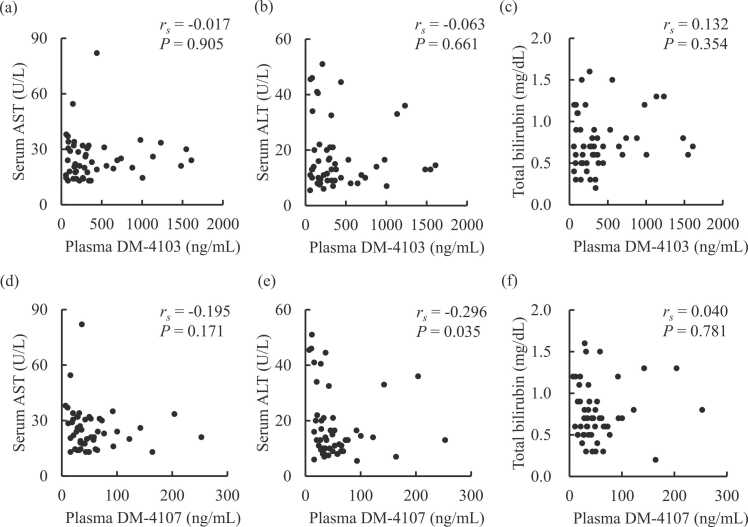
Correlations between serum liver function test results and plasma concentrations of tolvaptan metabolites. Plasma DM-4103 versus serum (a) AST, (b) ALT, and (c) total bilirubin; and plasma DM-4107 versus serum (d) AST, (e) ALT, and (f) total bilirubin. The correlations were evaluated using the Spearman test. ALT, alanine aminotransferase; AST, aspartate aminotransferase.

Table 2 shows the serum liver and biliary function test results before and after tolvaptan treatment. Although being within the normal ranges, significant decreases in serum AST (*P* = 0.002), ALT (*P* = 0.039), and total bilirubin (*P* = 0.006) were observed after tolvaptan administration. Tolvaptan treatment also resulted in decreased serum lactate dehydrogenase (*P* < 0.001), while the other liver and biliary function test results remained unchanged. As shown in Fig. 1, the plasma concentration of tolvaptan had significant negative correlations with serum AST (*r*_*s*_ = −0.341, *P* = 0.014), ALT (*r*_*s*_ = −0.410, *P* = 0.003), and total bilirubin (*r*_*s*_ = −0.358, *P* = 0.010). The plasma DM-4107 concentration also showed a negative correlation with serum AST (*r*_*s*_ = −0.296, *P* = 0.035) (Fig. 2). Plasma concentrations of tolvaptan and its metabolites exhibited no other associations with serum liver or biliary function test results.

**Table 2:** Serum liver and biliary function test results before and after tolvaptan treatment.

	Before	After	*P*-value
Aspartate aminotransferase, U/L	27 (21–37)	23 (18–31)	0.002
Alanine aminotransferase, U/L	17 (12–27)	13 (10–21)	0.039
Lactate dehydrogenase, U/L	252 (221–319)	220 (190–292)	< 0.001
Alkaline phosphatase, U/L	269 (212–300)	262 (225–325)	0.893
Leucine aminopeptidase, U/L	52 (43–63)	53 (46–62)	0.224
γ-glutamyl transpeptidase, U/L	36 (18–74)	32 (19–71)	0.572
Total bilirubin, mg/dL	0.70 (0.60–1.00)	0.70 (0.50–0.90)	0.006
Cholinesterase, U/L	200 (163–234)	193 (146–239)	0.746
Total protein, g/dL	6.6 (6.0–7.0)	6.5 (6.0–7.1)	0.338
Albumin, g/dL	3.6 (3.2–3.9)	3.5 (3.2–3.8)	0.483

Data are expressed as median with interquartile range in parentheses. All statistics were analyzed using the Wilcoxon signed-rank test.

### 3.4 Relationships with plasma biomarkers of OATP activity

**Fig. 3:**
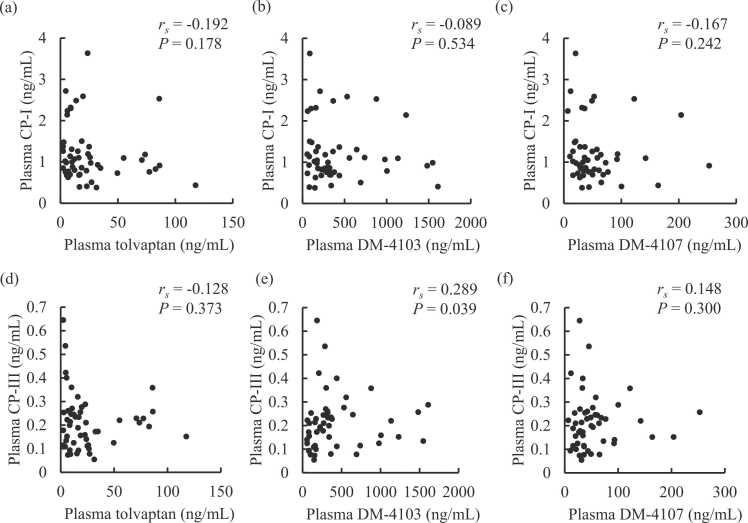
Correlations of plasma CP-I and CP-III with plasma concentrations of tolvaptan and its metabolites in heart failure patients. Plasma CP-I versus (a) tolvaptan, (b) DM-4103, and (c) DM-4107; and plasma CP-III versus (d) tolvaptan, (e) DM-4103, and (f) DM-4107. The correlations were evaluated using the Spearman test. CP-I, coproporphyrin-I; CP-III, coproporphyrin-III.

The median and IQR for plasma concentrations of CP-I and CP-III were 1.00 and 0.76–1.34, and 0.199 and 0.125–0.254 ng/mL, respectively. Seven patients had plasma concentrations of CP-III below the LLOQ, which were substituted by the LLOQ value. The median and IQR for DHEA-S were 0.458 and 0.139–0.691, respectively. Plasma CP-I (*r*_*s*_ = 0.559, *P* < 0.001) and CP-III (*r*_*s*_ = 0.296, *P* = 0.035) had positive correlations with serum total bilirubin, while plasma DHEA-S did not (*r*_*s*_ = −0.023, *P* = 0.875). No association was observed between plasma CP-I and CP-III (*r*_*s*_ = 0.156, *P* = 0.276). Plasma DHEA-S was correlated with neither plasma CP-I nor CP-III. Fig. 3 shows that the plasma concentration of DM-4103 had a positive correlation with plasma CP-III (*r*_*s*_ = 0.289, *P* = 0.039) but not with plasma CP-I (*r*_*s*_ = −0.089, *P* = 0.534). The plasma concentrations of tolvaptan and DM-4107 were not correlated with plasma CP-I and CP-III. The plasma concentrations of tolvaptan and its metabolites did not correlate with plasma DHEA-S.

### 3.5 Influence of transporter polymorphisms on plasma biomarkers of OATP activity

The genotype distributions of OATP1B1, OATP1B3, and ABCB1 polymorphisms are summarized in Table 3. The allele frequencies estimated based on the principle of the Hardy-Weinberg equilibrium were 66.7 %, 7.8 %, and 7.8 %; 69.6 %, 70.6 %, and 14.7 %; and 40.2 % for the genetic variants of *OATP1B1 388 A>G*, *T > C*, and *-11187G>A*; *T > G*, *699 G>A*, and *1683–5676 A>G*; and *ABCB1 3435 C>T*, respectively. Complete concordance was observed in the genotype distributions of *T > C* and *-11187G>A*. The combined corresponding rate of *T > G* and *699 G>A* also reached 98.0 %. Table 3 shows there was no difference in plasma CP-I between the respective OATP1B1, OATP1B3, and ABCB1 genotypes, while the patients with *OATP1B3 1683–5676AG* had lower serum total bilirubin than those with *OATP1B3 1683–5676AA* (*P* = 0.035). The genotypes analyzed did not show any other relationships with serum total bilirubin, plasma CP-I, CP-III, or DHEA-S.

**Table 3:** Plasma coproporphyrin-I and serum total bilirubin between respective genetic variants and genotype distributions of OATP1B1, OATP1B3, and ABCB1 polymorphisms.

(A) Plasma concentration of coproporphyrin-I (ng/mL)				
Genotype	Wild homozygote	Heterozygote	Mutant homozygote	*P*-value
*OATP1B1c.388 A>G*	1.28 (1.01–1.64)	0.86 (0.76–1.01)	1.07 (0.69–1.48)	0.094
OATP1B1c.521T > C *	0.98 (0.74–1.28)	1.19 (0.83–2.25)	- #	0.277
*OATP1B3c.334T > G*	0.85 (0.74–0.86)	1.09 (0.77–1.48)	0.98 (0.78–1.26)	0.410
*OATP1B3c.699 G>A*	0.85 (0.74–0.86)	1.10 (0.78–1.48)	0.98 (0.74–1.26)	0.334
*OATP1B3c.1683–5676 A>G*	0.98 (0.77–1.26)	1.05 (0.78–1.83)	- #	0.432
*ABCB1c.3435 C>T*	1.07 (0.77–1.37)	0.98 (0.73–1.67)	0.86 (0.81–1.18)	0.732
(B) Serum level of total bilirubin (mg/dL)				
Genotype	Wild homozygote	Heterozygote	Mutant homozygote	*P*-value
*OATP1B1c.388 A>G*	0.65 (0.50–0.83)	0.70 (0.60–0.80)	0.70 (0.50–0.90)	0.818
OATP1B1c.521T > C *	0.70 (0.50–0.90)	0.80 (0.70–1.13)	- #	0.106
*OATP1B3c.334T > G*	0.70 (0.60–0.80)	0.70 (0.60–1.20)	0.70 (0.50–0.80)	0.680
*OATP1B3c.699 G>A*	0.70 (0.60–0.80)	0.70 (0.60–1.20)	0.70 (0.50–0.80)	0.420
*OATP1B3c.1683–5676 A>G*	0.70 (0.60–1.13)	0.50 (0.45–0.75)	- #	0.035
*ABCB1c.3435 C>T*	0.70 (0.50–0.90)	0.60 (0.50–0.75)	0.80 (0.60–1.25)	0.389
(C) Genotype distributions of OATP1B1, OATP1B3, and ABCB1 polymorphisms				
Genotype	Wild homozygote	Heterozygote	Mutant homozygote	Allele frequency
*OATP1B1c.388 A>G*	8	18	25	66.7 %
*OATP1B1c.521T > C*	43	8	0	7.8 %
*OATP1B1 g.-11187G>A*	43	8	0	7.8 %
*OATP1B3c.334T > G*	3	25	23	69.6 %
*OATP1B3c.699 G>A*	3	24	24	70.6 %
*OATP1B3c.1683–5676 A>G*	36	15	0	14.7 %
*ABCB1 c.3435 C>T*	21	19	11	40.2 %

Data are expressed as the median with interquartile range in parentheses. All statistics were evaluated using the Kruskal-Wallis test or the Mann-Whitney *U* test.

* : Genotype distributions of *OATP1B1 g.–11187G>A* were completely the same as those of *OATP1B1c.521T > C*.

#: No patient had mutant homozygote of *OATP1B1c.521T > C* or *OATP1B3 c.1683–5676 A>G*.Data are expressed as the number of patients. The allele frequencies were estimated based on the principle of the Hardy-Weinberg equilibrium.

#: No patient had mutant homozygote of *OATP1B1c.521T > C* or *OATP1B3c.1683–5676 A>G*.

### 3.6 Relationships with plasma biomarkers of drug-induced liver injury

The median and IQR for the plasma concentration of K18 were 38.6 and 24.2–61.2 pg/mL, respectively. The corresponding values for GLDH were 4.4 and 2.9–5.6 U/L, where the data of plasma GLDH were replaced by 1.0 U/L for 3 patients who had a value below the LLOQ. Plasma K18 had positive correlations with serum AST (*r*_*s*_ = 0.338, *P* = 0.015) and ALT (*r*_*s*_ = 0.420, *P* = 0.002), but not with total bilirubin (*r*_*s*_ = −0.039, *P* = 0.788). Plasma GLDH had no associations with serum AST, ALT, and total bilirubin. No association was observed between plasma K18 and GLDH (*r*_*s*_ = −0.109, *P* = 0.445). The plasma concentrations of tolvaptan and its metabolites were not correlated with plasma K18 and GLDH.

## 4 Discussion

This study found that the plasma concentration of tolvaptan was inversely correlated with AST, ALT, and T-Bil in heart failure patients (Fig. 1). These laboratory data improved after tolvaptan administration (Table 2). DM-4107 was significantly correlated with ALT (Fig. 2), while DM-4103 was not. Tolvaptan may achieve liver protection directly or via inhibition of drug transporters. Tolvaptan may cause liver injury [5]. The result of this current study seems to indicate an inverse effect of tolvaptan on liver function. This study also investigated the relationship of tolvaptan and its metabolites to CP-I and CP-III. Significant correlation was found between DM-4103 and CP-III (Fig. 3).

Patients receiving low dose tolvaptan (3.75–15 mg daily) were included. As a result, low concentrations of tolvaptan and its metabolites were observed. In this concentration range, the tolvaptan concentration was correlated with AST, ALT, and T-Bil. DM-4103 did not correlate with AST, ALT, and T-Bil. The half-maximal inhibitory concentration of DM-4103 for OATP1B1 was reported to be 0.255 µM [7] (= 122 ng/mL). The interquartile plasma concentration range of DM-4103 was 157–485 ng/mL in this study. Considering that its protein binding rate is 99.8 %, the DM-4103concentration seems too low to inhibit OATP1B1. DM-4107 was significantly correlated only with ALT. Therefore, DM-4103 and DM-4107 have a smaller effect on liver laboratory values than tolvaptan in clinical settings. Tolvaptan may affect liver function directly. K18 did not correlate with the plasma concentrations of tolvaptan and its metabolites. K18 was not correlated with GLDH. K18 and GLDH have been reported to be markers of drug induced liver injury [16], [17]. K18 reflects cytoskeleton injury [16] and GLDH reflects mitochondrion injury [17]. No significant differences in K18 and GLDH were observed between OATP genotypes. They are independent from OATP activity. The effect of tolvaptan is independent of cytoskeleton and mitochondrion injury [19].

The serum concentration of bilirubin was lower in the patients with *OATP1B3c.1683–5676 A>G* compared to those with the wild homozygote (Table 3). Bilirubin protects hepatocytes through antioxidative action. Bilirubin is a substrate for ABCB1, and it is also uptaken into hepatocytes by OATP1B1 and OATP1B3 [2]. Interaction with OATP may affect liver function via bilirubin intake. Tolvaptan may decrease bilirubin in hepatocytes by inhibiting OATP1B1 and OATP1B3. A decrease in bilirubin in hepatocytes can easily cause liver injury. Tolvaptan and its metabolites did not correlate with CP-I, CP-III, and DHEA-S in this study. CP-I and CP-III are substrates of OATP1B1 and OATP1B3. Serum CP is an indicator of OATP activity [10], [11] and DHEA-S can be an indicator of NTCP activity [20]. DHEA-S is also an indicator of OATP activity. CP-I and CP-III are independent from liver injury and more selective indicators for OATP activity than bilirubin. No significant relationships were found between DHEA-S and tolvaptan and its metabolites in this study. Tolvaptan did not affect OATP and NTCP activity.

The major limitation of this study is the comedication of a statin and amiodarone. Statins are a substrate of OATP and ABCB1 [21]. Amiodarone inhibits ABCB1 [22]. They may interact with the tolvaptan and endogenous CP-I and CP-III. Their dosages were constant and the effects of the interaction seem to also be stable. This study did not evaluate CP-I and CP-III before tolvaptan administration. Comparison of them between before and after administration might clarify the drug interaction. We observed improvements in AST and ALT after tolvaptan administration and the improvements were concentration dependent (Fig. 1). However, these laboratory values seemed to be less specific for liver function. AST and ALT were reported to be low in renal failure patients with liver injury [23]. The present study excluded hemodialysis patients. LDH improved after tolvaptan administration (Table 2), which may mainly reflect heart function rather than liver function. Improvements in AST and ALT may be caused by improvement of hepatic congestion. Tolvaptan efficacy is concentration-dependent, so the correlation in Fig. 1 is reasonable. However, AST and ALT are lower before administration and the effect of hepatic congestion may be limited. Careful interpretation of the present findings is needed.

In conclusion, low dose tolvaptan may ameliorate liver function in heart failure patients. The improvement of liver function after tolvaptan administration seems to be independent from serum bilirubin and OATP activity. The specific mechanism underlying the improvement of liver function was not determined so further study on the relationship between tolvaptan and liver function using a different approach is warranted.

## Funding

This research did not receive any specific grants from funding agencies in the public, commercial, or not-for-profit sectors.

## Author contributions statement

KH and TN planned and designed this study. Acquisition of data was carried out by KH, SA, and MS. YM, KH, TN, and JK contributed to the analysis and interpretation of data. All authors contributed to drafting and revision of the manuscript for important intellectual content and provided final approval for publication.

## CRediT authorship contribution statement

**Imoto Yumi:** Methodology, Investigation. **Akutsu Shunta:** Investigation, Data curation. **Naito Takafumi:** Supervision, Conceptualization. **Hoshikawa Kohei:** Data curation, Conceptualization. **Mino Yasuaki:** Writing – review & editing, Writing – original draft, Project administration. **Kawakami Junichi:** Supervision, Project administration. **Maekawa Yuichiro:** Supervision, Project administration. **Saotome Masao:** Resources, Data curation. **Nakatsugawa Emi:** Methodology, Investigation.

## Declaration of Competing Interest

The authors declare that they have no known competing financial interests or personal relationships that could have appeared to influence the work reported in this paper.

## Data Availability

Data will be made available on request.
